# Light-regulated voltage-gated potassium channels for acute interrogation of channel function in neurons and behavior

**DOI:** 10.1371/journal.pone.0248688

**Published:** 2021-03-23

**Authors:** Henry H. Jerng, Jay M. Patel, Tamor A. Khan, Benjamin R. Arenkiel, Paul J. Pfaffinger

**Affiliations:** 1 Departments of Neuroscience, Baylor College of Medicine, Houston, Texas, United States of America; 2 Molecular & Human Genetics, Baylor College of Medicine, Houston, Texas, United States of America; Doheny Eye Institute/UCLA, UNITED STATES

## Abstract

Voltage-gated potassium (Kv) channels regulate the membrane potential and conductance of excitable cells to control the firing rate and waveform of action potentials. Even though Kv channels have been intensely studied for over 70 year, surprisingly little is known about how specific channels expressed in various neurons and their functional properties impact neuronal network activity and behavior *in vivo*. Although many *in vivo* genetic manipulations of ion channels have been tried, interpretation of these results is complicated by powerful homeostatic plasticity mechanisms that act to maintain function following perturbations in excitability. To better understand how Kv channels shape network function and behavior, we have developed a novel optogenetic technology to acutely regulate Kv channel expression with light by fusing the light-sensitive LOV domain of *Vaucheria frigida* Aureochrome 1 to the N-terminus of the Kv1 subunit protein to make an Opto-Kv1 channel. Recording of Opto-Kv1 channels expressed in *Xenopus* oocytes, mammalian cells, and neurons show that blue light strongly induces the current expression of Opto-Kv1 channels in all systems tested. We also find that an Opto-Kv1 construct containing a dominant-negative pore mutation (Opto-Kv1(V400D)) can be used to down-regulate Kv1 currents in a blue light-dependent manner. Finally, to determine whether Opto-Kv1 channels can elicit light-dependent behavioral effect *in vivo*, we targeted Opto-Kv1 (V400D) expression to Kv1.3-expressing mitral cells of the olfactory bulb in mice. Exposure of the bulb to blue light for 2–3 hours produced a significant increase in sensitivity to novel odors after initial habituation to a similar odor, comparable to behavioral changes seen in Kv1.3 knockout animals. In summary, we have developed novel photoactivatable Kv channels that provide new ways to interrogate neural circuits *in vivo* and to examine the roles of normal and disease-causing mutant Kv channels in brain function and behavior.

## Introduction

Optogenetic regulation of neuronal activity by the photo-activation of excitatory or inhibitory currents is one the most impactful experimental revolutions in neuroscience [1,2]. Light is typically used as an On- or Off-switch by stimulating intrinsically light-sensitive excitatory or inhibitory channels or pumps, and the subsequent regulation of neuronal firing allows researchers to examine how firing of specific neuronal populations might be relevant to a behavior under study. While this approach is very useful for driving or suppressing different neuronal populations, how the normal electrophysiological properties of these neurons relate to their role in shaping circuit function and behavior remains poorly understood. Therefore, we set out to develop a new optogenetic tool to probe how voltage-gated potassium channels operate in specific neurons to influence circuit function and behavior.

Voltage-gated potassium (Kv) channels are important regulators of neuronal excitability and thus represent ideal targets for optogenetic control [3]. A previous approach to optically regulate Kv channels involves photoactivating a tethered blocker to a heterologously expressed Kv channel with an appropriately engineered cysteine near the pore (SPARK channel) [1,4]. An advantage of this system is that the photoswitch is fast and reversible; however, implementation of this tool faces the challenges of properly activating and controlling the expression of the SPARK, and efficiently modifying the cysteine in vivo [5].

To better understand how changes in Kv currents regulate neuronal activity and behavior, we sought to create a Kv channel construct that would allow optogenetic control of functional channel expression via manipulations of the endogenous cellular mechanism involved in channel assembly, without requiring a post-translational chemical modification reaction. Kv channels are formed via tetrameric assembly of alpha-subunits that belong to the same subfamily. Their N-terminal tetramerization (T1) domains play essential roles in channel assembly and subfamily specificity [6–8]. Deletions or mutations in the T1 domain can alter the ability of Kv channel subunits to assemble and thus fail to form functional channels [9,10]. Incompletely formed potassium channel complexes or unassembled subunits are typically strongly retained in the endoplasmic reticulum (ER) and shunted to degradation pathways rather than to the cell surface [11–16].

To regulate assembly of Kv channels with light, we fused a LOV (Light-Oxygen-Voltage) domain into the channel sequence adjacent to the N-terminus of the T1 domain. LOV domains are photoswitches from plant and unicellular organisms that are triggered by the absorption of blue light by a flavin mononucleotide (FMN) co-factor sterically linked to various effector domains [17–23]. Blue light induces the formation of a covalent adduct between a bound FMN and a cysteine residue (C4a), which transduces a signal to the effector domains via allosteric regulation of N- (A’-alpha) and C-terminal (J-alpha) helices [24]. Upon returning to the dark, the adduct state spontaneously decays to an oxidized flavin as the dark state is restored. Because FMN is found in all cells, LOV domains can be readily transferred to other proteins to engineer light-sensitivity and permit light-induced regulation of different cellular functions.

To create an optogenetically regulated Kv1 channel (Opto-Kv1) we fused the *Vaucheria frigida* aureochrome1 (VfAu1) LOV domain to the Aplysia Kv1 (aKv1) subunit protein at a position just N-terminal to the T1 domain [25]. As a member of the Kv1 subfamily, aKv1 is highly homologous to mammalian Kv1 channels, and the aKv1 T1 domain contains assembly interfaces that are identical to almost all other vertebrate and invertebrate Kv1 subunits, well characterized functionally, and distinct and easily followed in mammalian systems [8]. We find that Opto-aKv1 channels exposed to blue light show a dramatic increase in functional current, while retaining the normal subfamily-specific assembly and functional properties of the wild-type channel. Opto-aKv1 channels containing a dominant-negative pore mutation (V400D) produce light-dependent suppression of wild-type Kv1 channels in mammalian cells and neurons, and when Opto-aKv1(V400D) is expressed in mitral cells of the olfactory bulb, blue light exposure induces measurable olfactory behavioral changes that are similar to what is seen with the Kv1.3 knockout.

## Results

### Rational design of Opto-Kv channels

**Fig 1:**
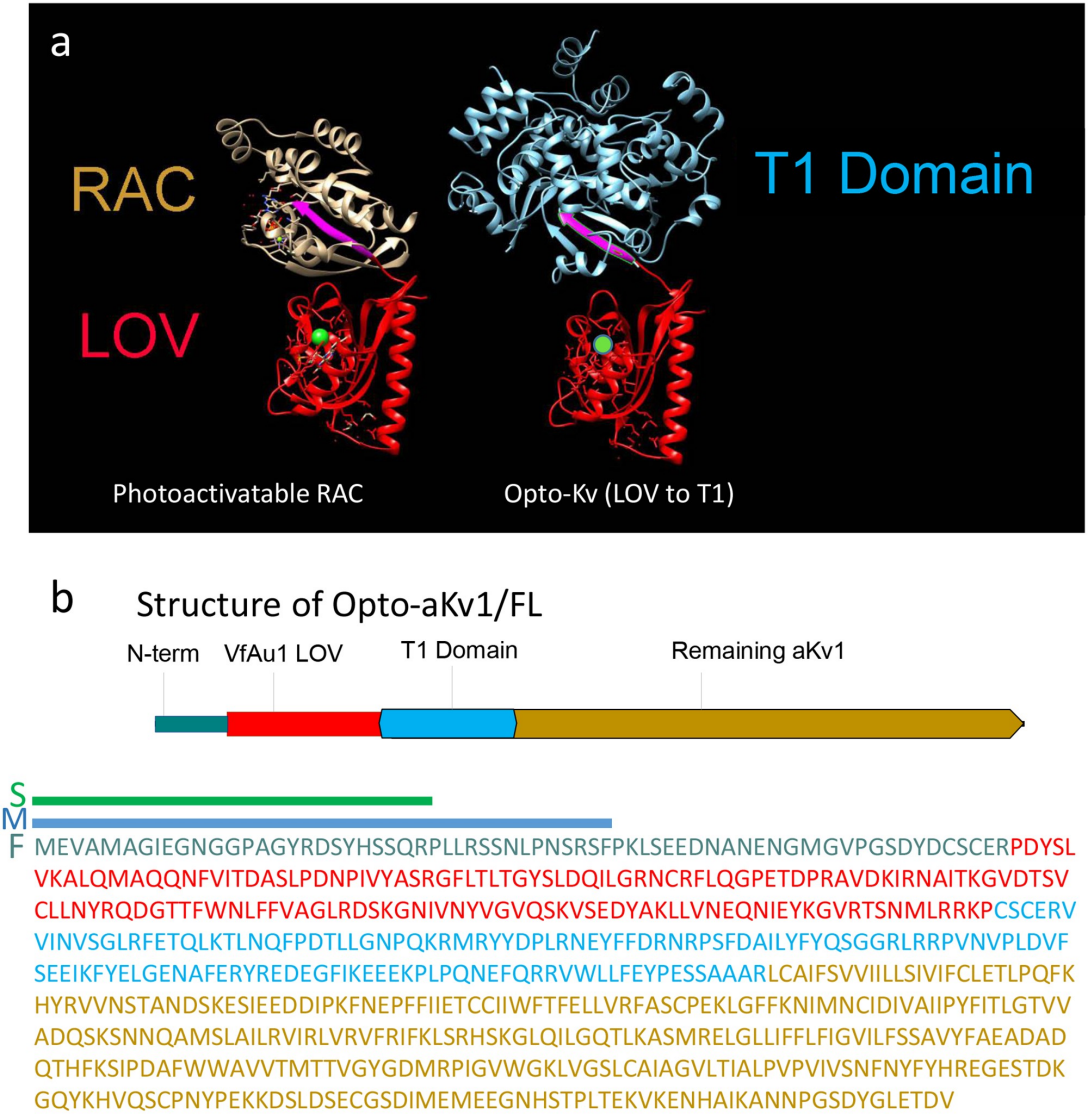
Rationale and design of Opto-Kv channels. **a**) Structural design mapping a photoactivatable derivative of Rac1 (LOV-Rac) onto a Opto-Kv LOV-T1 fusion domain. (Left) Crystal structure of a photoactivatable Rac1 containing wild-type LOV2 (Protein Data Bank 2WKP). It shows the connection of LOV domain J-alpha helix (red) to the N-terminal beta strand of Rac1 (magenta). (Right) The LOV2 domain was mapped by homology onto the N-terminal beta strand of aKv1 T1 domain (magenta) (Protein Data Bank 1T1D). The T1 domain was tilted 45°C off axis to show the linkage similarity. A proline residue was introduced into the LOV-T1 linker to move the LOV domain into a better position to disrupt T1-T1 interactions. **b**) Domain structure and sequence margins for Opto-Kv channel constructs using aKv1 channel. Residue color matches that of the corresponding domain. N-terminal amino acids included in the Opto-aKv1/SL (green) and Opto-aKv1/ML (blue) variants are indicated by the colored bars.

LOV domains have been used to generate a wide variety of light-regulated biological switches to turn on or off biological processes with light [17,26]. The LOV domain from *Vaucheria frigida* Auerochrome 1 has been used to control protein-protein interactions to create, for example, light-regulated receptor tyrosine kinases, transcription factors, or a light-regulated protease casp9 [27–29]. To create Opto-Kv channels, we fused the VfAu1 LOV domain (aa 204–348) to the N-terminus of the T1 domain of the inactivating Kv1 voltage-gated potassium channel from Aplysia (Fig 1) [8,25]. This fusion site was chosen to minimize the potential impact of the LOV domain on native channel properties, since the regions C-terminal to the T1 domain (including the voltage-sensor and pore-forming regions) are involved in voltage-dependent gating and potassium conduction. In our initial construct, we retained the aKv1 N-type inactivation domain N-terminal to the LOV domain to test potential impacts of this fusion on N-type inactivation, creating Opto-aKv1/FL (full-length). We hypothesized that placing the C-terminal J-alpha helix of the LOV domain next to the first beta strand of the T1 domain may induce light-mediated regulation by either (1) sterically impeding T1 tetramerization or by (2) modulating the structure of the first beta strand of the T1 domain in a manner similar to LOV domain regulation of RAC1 (Fig 1a) [30].

### Blue light induces functional expression of Opto-Kv channels

**Fig 2:**
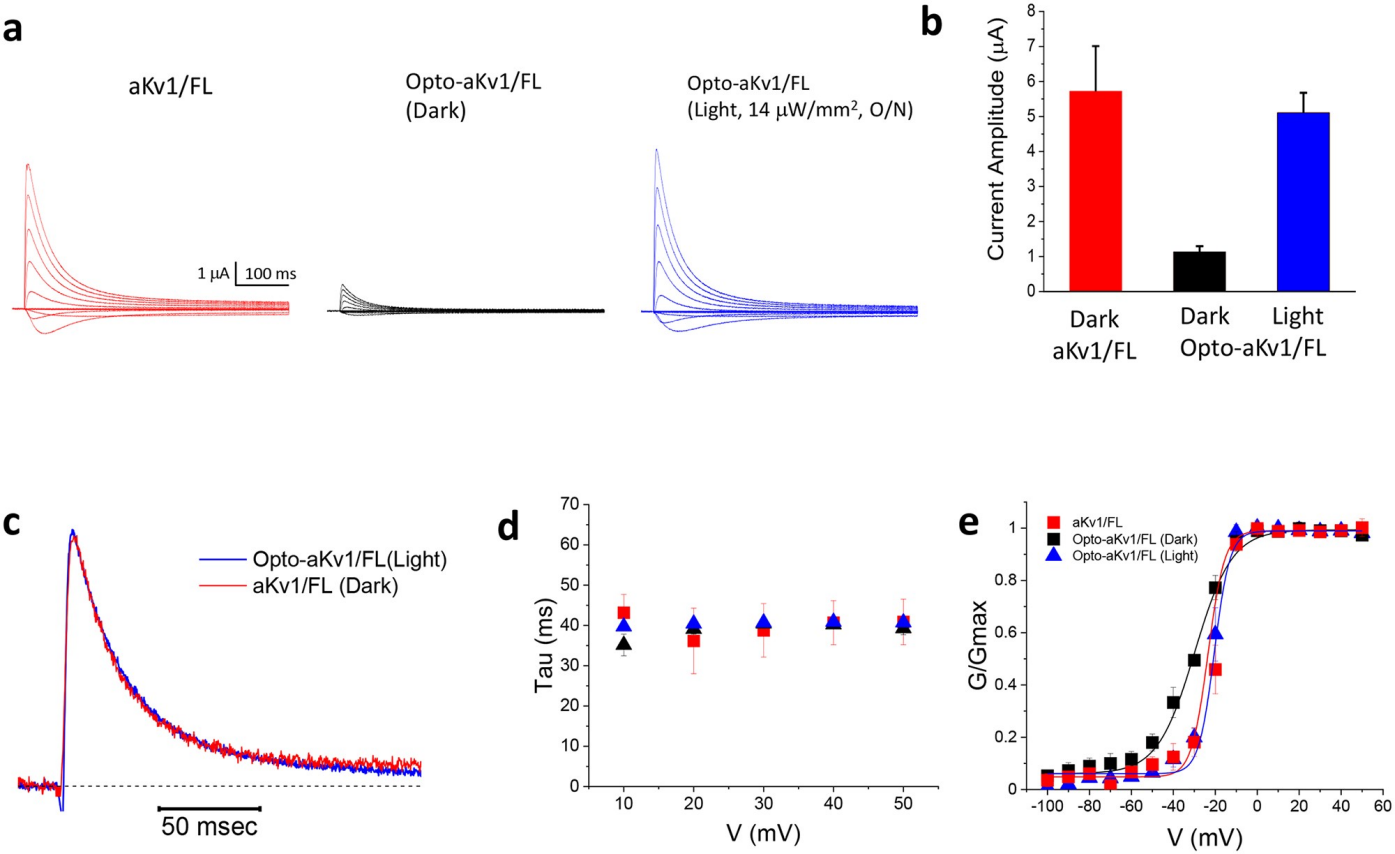
Channel expression suppressed by LOV domain and its photoactivation by light. **a**) Currents in *Xenopus* oocytes following injection of various cRNAs (aKv1/full-length and Opto-aKv1) and overnight expression at 18°C under dark or blue light conditions. Traces were elicited by voltage steps from -60 mV to +50 mV at 10 mV steps from a holding potential of -100 mV. Blue light was applied continuously for 16 hrs at 14 μW/mm^2^. **b**) Analysis of peak current amplitude at +50 mV. Results plotted as mean ± standard error (SE) (p < 0.01 Dark-Light). **c**) Close-up comparison of current kinetics for Opto-aKv1/FL with blue light and aKv1/FL in the dark recorded at +50 mV using elevated K+ Out. Following normalization for peak current amplitude the current waveforms are nearly identical. **d**) Inactivation of outward currents as measured using single exponential decay at described voltages. **e)** Conductance-voltage relationship of aKv1/FL channels compared to those of Opto-aKv1/FL in the dark or light. Results are shown as mean ± SEM.

To first test these constructs, wild-type AKv1 and Opto-aKv1 channels were heterologously expressed in *Xenopus* oocytes by cRNA injections, and whole-oocyte currents were recorded using the two-electrode voltage clamp technique. Upon prolonged depolarization, wild-type aKv1 channels express large outward potassium currents that undergo rapid N-type and slow C/P-type inactivation (Fig 2a). Because N-type inactivation accelerates C/P-type inactivation that recovers slowly, wild-type aKv1/FL and Opto-aKv1/FL currents were recorded with an elevated external K+ concentration of 98 mM to slow entry into and speed recovery from C-type inactivation during repetitive depolarizing pulses [31]. Elevating external K+ concentration to 98mM from 2 mM results in a potassium reversal potential of approximately 0 mV. When oocytes expressing Opto-aKv1/FL channels were tested after overnight incubation in the dark, the expressed currents were small (~1 μA) in spite of varying the amount of cRNA injected in the range of 2 to 8 ng/oocyte (Fig 2a). Compared to wild-type aKv1/FL channels expressed under the same conditions, Opto-aKv1/FL currents were reduced 6-fold (Fig 2b). To examine whether this reduction in expressed current was sensitive to light, oocytes injected with Opto-aKv1/FL cRNA were exposed to blue light (14.2 μW/mm^2^) overnight prior to recording. Results showed that blue light application fully rescued the Opto-aKv1/FL outward currents, with amplitudes of Opto-aKv1/FL currents with overnight light treatment being similar to wild-type aKv1/FL currents expressed in the dark (Fig 2a & 2b). These results indicate that the insertion of LOV domain at the N-terminus of the T1 domain led to reduced surface expression in the dark, and that blue light reverses the inhibitory effects of the LOV domain (Fig 2b).

We noted that photoactivated Opto-aKv1/FL channels express at similar levels as wild-type aKv1/FL channels, but we also sought to determine if they are functionally equivalent. Comparison of channel current waveforms at +50 mV showed that the kinetics of activation and inactivation of aKv1/FL were indistinguishable from wild-type aKv1/FL following light induction (Fig 2c). The inactivation time constants at all positive potentials were not significantly different between aKv1/FL, Opto-aKv1/FL (dark), and Opto-aKv1/FL (light) (Fig 2d). In addition, the conductance-voltage relationship for activation of wild-type aKv1/FL is well described with a Boltzmann function having a midpoint of -19.8 ± 1.7 mV and a slope of 4.4 ± 1.5 mV/e-fold (n = 3). Following overnight exposure to blue light, the induced Opto-aKv1/FL channel activation midpoint (-21.8 ± 0.8 mV) and activation slope (4.48 ± 0.40 mV/e-fold; n = 4) were indistinguishable from wild-type channels (Fig 2e). Therefore, the biophysical properties of the photoactivated Opto-aKv1/FL channels do not appear to be different from those of wild-type aKv1/FL channels. Interestingly, the conductance-voltage relationship plot shows the small, residual Opto-aKv1/FL current that expressed in the dark appeared to activate at more hyperpolarized potentials with less voltage sensitivity (activation midpoint = -29.9 ± 0.75 mV, activation slope = 8.91 ± 0.43 mV/e-fold, n = 4) (Fig 2e). This result suggests that the few Opto-aKv1/FL channels that emerged on the oocyte surface in the dark are functionally distinct, possibly because of unusual T1 domain tetramerization.

**Fig 3:**
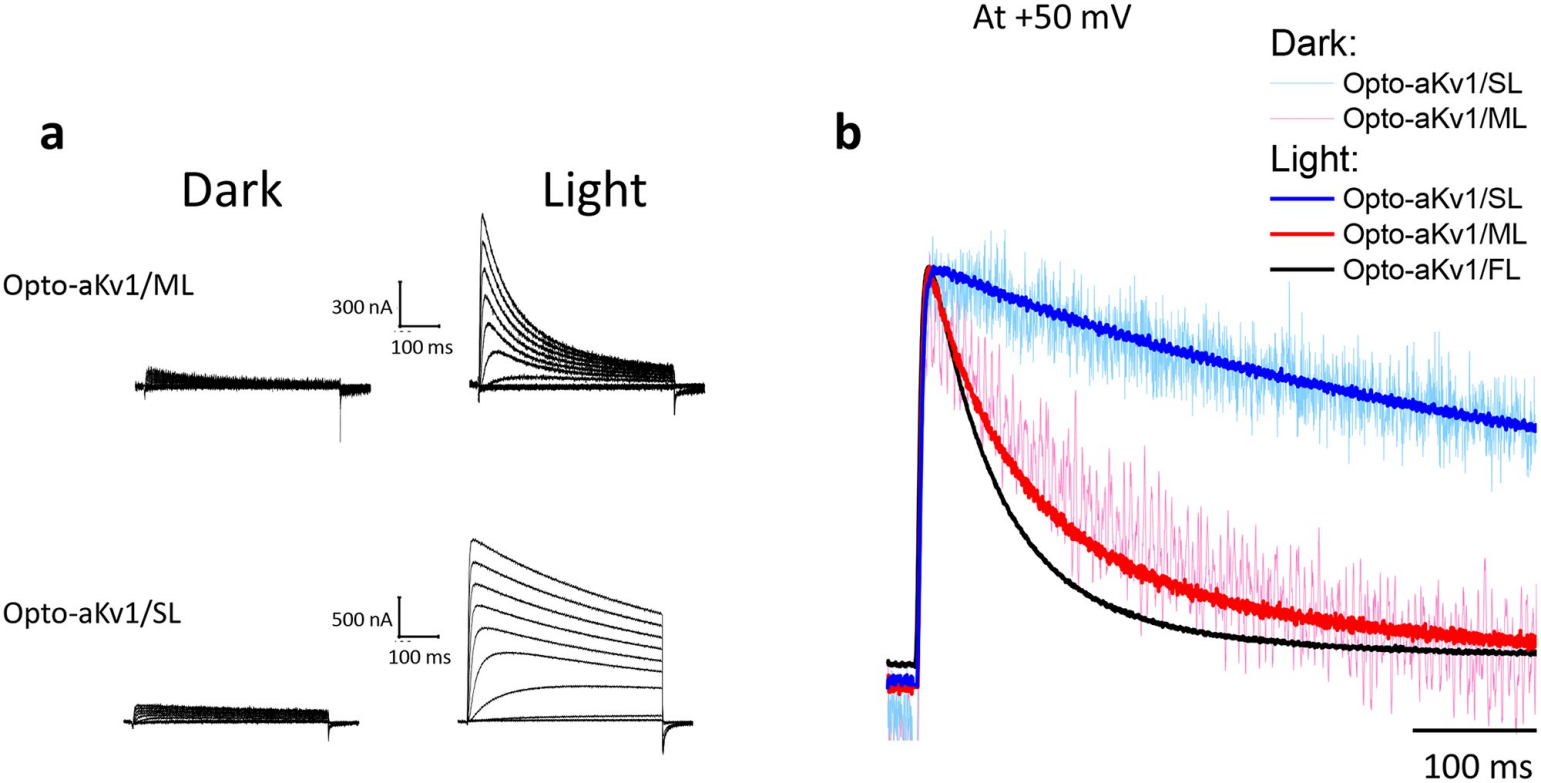
N-terminal variants of Opto-aKv1 show different inactivation kinetics but similar levels of induction with blue light. **a)** Outward currents expressed by Opto-aKv1/ML and Opto-aKv1/SL elicited by depolarizing pulses as described in Fig 2a. Application of blue light, as observed with Opto-aKv1/FL, dramatically induced surface expression of Opto-aKv1/ML and Opto-aKv1/SL. **b)** Overlapped traces of Opto-aKv1/FL, Opto-aKv1/ML, and Opto-aKv1/SL at +50 mV with blue light treatment.

Fig 1a shows that N-type inactivation of Opto-aKv1/FL appeared to be completely normal and exhibited no light sensitivity, suggesting that neither the insertion of the LOV domain, nor the light-dependent conformational changes of the LOV domain affect access of the N-terminal ball peptide to the pore region of the channel. Given this, we next engineered additional N-terminal Opto-aKv1 variants with progressively shorter chain sequences, including Opto-aKv1/ML (medium length) and Opto-aKv1/SL (short length) variants (see Fig 1b) to (1) evaluate whether variations in N-terminal sequences impact light-mediated induction, and (2) vary N-type inactivation and test whether shorter chains produced light sensitive N-type inactivation. Opto-aKv1/ML and Opto-aKv1/SL constructs were expressed and tested in oocytes, and similar to Opto-aKv1/FL, these channels expressed very poorly in the dark and strongly responded to overnight blue light exposure with a marked 8- to 10-fold induction (at +50 mV: Opto-aKv1/ML (dark) = 0.144 ± 0.03 uA, n = 3; Opto-aKv1/ML (light) = 1.42 ± 0.18 uA, n = 3; Opto-aKv1/SL (dark) = 0.23 ± 0.02 uA, n = 3; Opto-aKv1/SL (light) = 1.90 ± 0.57 uA, n = 3) (Fig 3a). Their corresponding currents under normal external K+ concentration showed progressively slower inactivation kinetics as the N-terminus was shortened from Opto-aKv1/ML to Opto-aKv1/SL, but also no effects of light on their inactivation kinetics (Fig 3b). Together, these data confirm the utility of Opto-aKv1/FL modulation of K+ currents, and demonstrate that variations in the structure of the construct N-terminal to the LOV domain do not affect the light-dependent regulation of the Opto-Kv construct, affording the construction of designer photoswitchable Kv channels with a variety of kinetic properties.

### Time course of Opto-Kv channel induction by light

**Fig 4:**
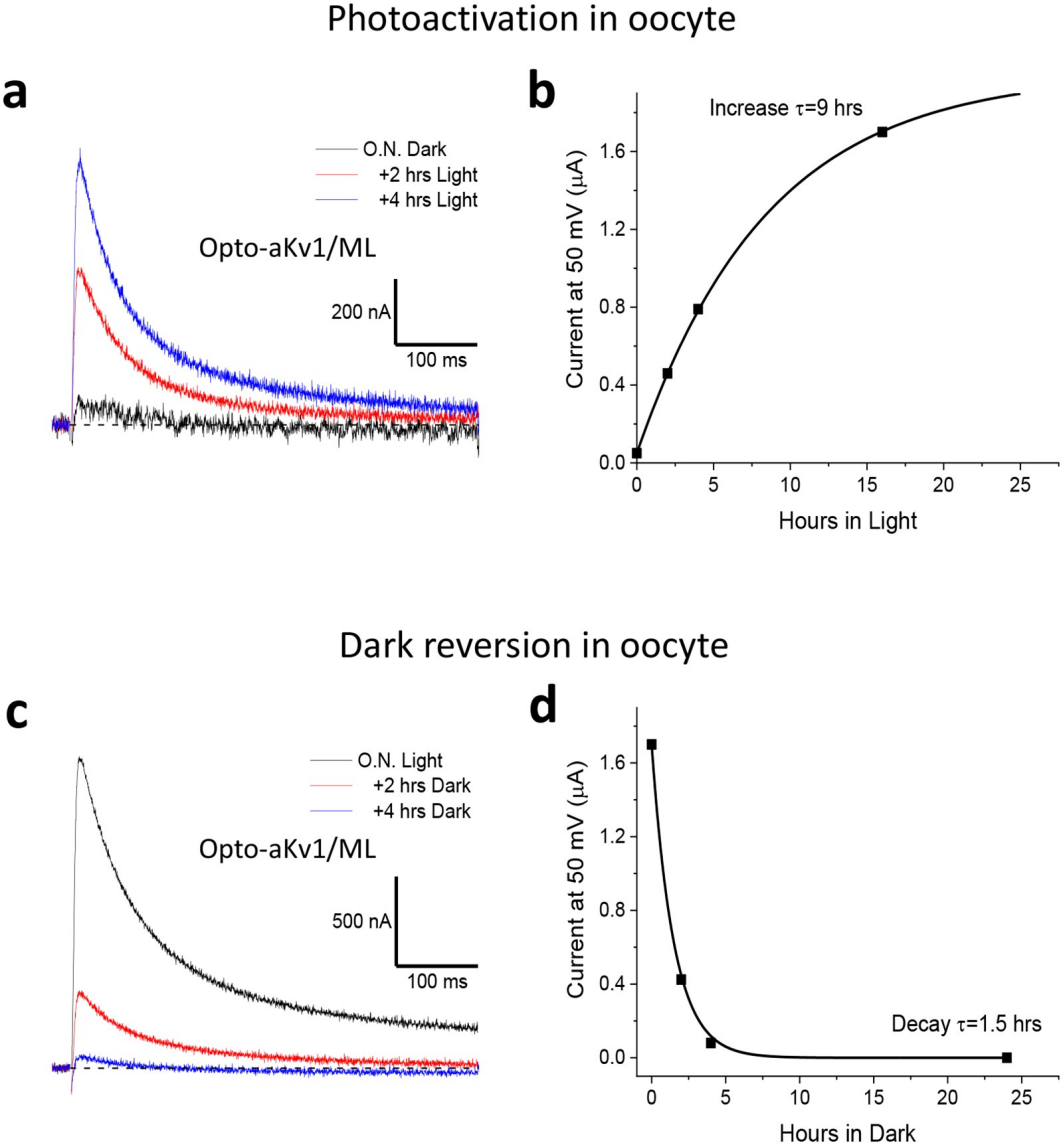
Time course of photoactivation and deactivation in oocytes. Opto-aKv1/ML channels were expressed in *Xenopus* oocytes overnight in the dark or with blue light exposure. Currents were elicited by +50 mV depolarization from -100 mV holding potential. **a**) Photoactivation of current. Oocytes incubated in the dark overnight expressed very little current; however, progressively longer exposure to blue light led to increasing current expression. **b**) Time course of photoactivation in an individual oocyte. Induction of current by blue light is significant within 2 hours of exposure and follows a 9 hr time constant. **c**) Deactivation by darkness. Oocytes exposed to blue light overnight were left in the dark and then tested at the described time points. **d**) Single exponential fit to dark deactivation of induced current shows a 1.5 hr time constant to return to baseline.

The activation and deactivation for wild-type VfAU1-LOV, as measured by FMN-C4a adduct decay kinetics, occurs in a matter of minutes at 20°C [27,32,33]. To better understand the time course for light-mediated induction of Opto-Kv1, we used the same light stimulation paradigm, taking measurements of current amplitude at set time points from the same oocytes. For Opto-aKv1/ML, recordings show that photoactivation of current is evident within 2 hours after light exposure and continues to climb until plateauing at a functional level similar to wild-type aKv1 (Fig 4a). Upon returning to the dark, current loss was quicker, with most induced current decaying within 2 hours of light removal (Fig 4c). Changes in current levels followed an approximated single exponential time course in response to light application or removal. Single exponential fits to our data suggest a time constant of approximately 9 hours for light-dependent induction of current (Fig 4b), and a time constant of 1.5 hours for loss of current when shifted to the dark (Fig 4d). The slow time course by which current was induced and lost, and the lack of acute effects of light on channels that have reached the cell surface suggests channel assembly and surface trafficking kinetics are the main determinants of the time course for changes in functional channel levels.

### Rapid photoinduction and removal of Opto-aKv1 channels from the cell surface

**Fig 5:**
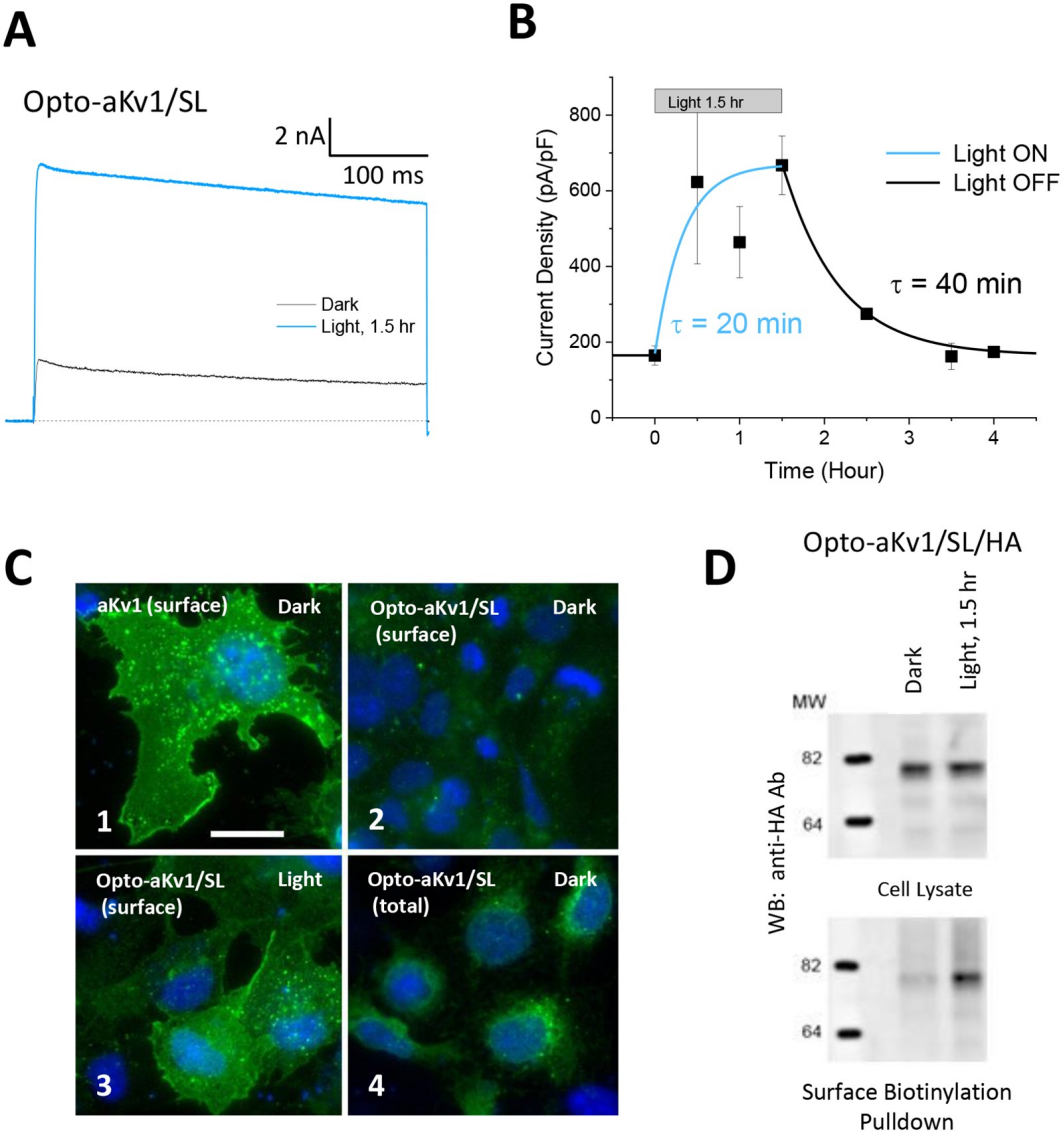
Rapid photoinduction and deactivation of Opto-aKv1 channels from mammalian cell surface membrane. **a**) Photoactivation of Opto-aKv1/SL channels by blue light treatment. Transfected HEK293K cells were incubated in the dark or under blue light for 1.5 hours before voltage-clamp recordings. Currents were elicited by depolarization to +50 mV from holding at -80 mV for 400 ms. Currents were also measured after returning the cells to dark for varying amount of time until the baseline was reached. **b**) Current density in response to light plotted as a function of time. The time course of both photoactivation and deactivation were significantly more rapidly in HEK293K cells than in oocytes. **c**) Visualization of wild-type aKv1 and Opto-aKv1 expression in the dark or under blue light by immunostaining. Total Opto-aKv1 protein was obtained by permeabilizing the cells before antibody staining; surface proteins, without permeabilization. Scale bar = 20 um. **d**) Confirmation of increase in surface expression in response to blue light treatment. CHO-K1 cells transfected with Opto-aKv1/SL/HA were left in the dark or treated with blue light for 1.5 hours. Surface Opto-aKv1/SL channels tagged with external HA epitope (Opto-aKv1/SL/HA) were labelled with biotin before cell lysis and pulldown using streptavidin beads. Proteins separated on Western blots were probed with anti-HA antibody (1:200).

Based on these results, we next expressed Opto-Kv channels in mammalian cells and tested for light-dependent functional expression at 37 °C. Photoinduction of Opto-aKv1/SL channels in HEK293 cells revealed dramatically faster responses than those observed for Opto-aKv1/SL channels in oocytes (Fig 5a). The photoinduction of Light-ON response occurred within 30 minutes, plateauing by 1.5 hours (Fig 5b). A single exponential fit to the data gave a Light-ON time constant of 20 minutes. Following a return to darkness, we also evaluated the Light-OFF responses (Fig 5b). Within 2 hours in the dark, the induced current completely dissipated at a rate described by a single exponential time constant of 40 minutes. These results suggest that both trafficking of channels to the surface and endocytosis of channels from the membrane are significantly faster in mammalian cells compared to oocytes, likely related to the higher incubation temperature for mammalian cells (37 °C) verses oocytes (18 °C) [34].

To confirm that the observed electrophysiological differences are due to a light-dependent regulation of Opto-Kv1 assembly and surface trafficking, as seen in oocytes, we performed immunocytochemistry and surface biotinylation experiments. Immunocytochemisty on non-permeabilized COS7 cells revealed high levels of surface expression of wild-type aKv1 channels in the dark (Fig 5c1), but not for Opto-Kv1/SL (Fig 5c2). After 1.5 hrs of blue light, Opto-Kv1 channel surface staining increased to wild-type levels (Fig 5c3). Permeabilized staining showed that in the dark, Opto-Kv1 subunits are present internally and were primarily restricted to peri-nuclear endoplasmic reticular membranes (Fig 5c4). Similar results were found using surface biotinylation pulldown assays to biochemically examine the amount of Opto-aKv1/SL protein on the surface and compare it to total Opto-aKv1/SL protein (Fig 5d). We found that, in the dark, only a small fraction of total Opto-aKv1/SL protein reached the surface, consistent with our recordings. The remaining Opto-aKv1/SL proteins showed intracellular localization consistent with the ER localization as seen in our immunofluorescence data. Following 1.5 hrs of blue light exposure, the amount of Opto-aKv1/SL proteins on the surface dramatically increased, in agreement with the electrophysiological recordings. Collectively, these data show that the LOV domain in the dark causes retention of Opto-Kv channel subunits inside the cell, and that blue light is capable of inducing conformational changes in the LOV domain that allow retained subunit proteins to be trafficked to the cell surface.

### Photoactivation of dominant negative Opto-aKv1 (Opto-aKv1/V400D) subunits suppresses wild-type aKv1 and native Kv1.3 currents

Since Opto-Kv1 subunits assemble functional channels and traffic to the surface following blue light exposure, we next sought to test if these subunits can be used to interact with and suppress wild-type Kv1 subunits in a light-dependent manner. To this end, we introduced a dominant-negative mutation from the Sh(KS133) *Shaker* mutant of *Drosophila* into the pore region of the Opto-aKv1/SL subunit (Opto-aKv1/SL/V400D) [35]. Opto-aKv1/SL subunits do not show fast N-type inactivation and therefore are kinetically distinct from fast inactivating wild-type aKv1 subunits, and so we can use inactivation kinetics along with current amplitude to monitor the dominant negative suppression of wild-type current. The prediction was that tetrameric channels that incorporate one or more V400D mutant subunit, rather than having slower inactivation kinetics, would become electrically silent due to the pore mutation eliminating potassium conductance. To first test this, we co-transfected CHO cells with both wild-type aKv1/FL and Opto-aKv1/SL/V400D constructs and incubated overnight at 37 °C in the dark. Whole-cell patch clamp recordings showed no outward K+ currents from untransfected cells but robust currents from cells co-transfected with aKv1/FL and Opto-aKv1/V400D (Fig 5a). The kinetics and amplitudes were similar to those with wild-type aKv1/FL subunits alone, indicating that Opto-aKv1 subunits do not effectively tetramerize with wild-type AKv1 subunits in the dark. When the co-transfected CHO cells were stimulated with blue light for 1.5 hours, the amplitude of the fast inactivating aKv1 current dramatically declined without changing inactivation kinetics, indicating that the dominant-negative subunits co-assembled with wild-type aKv1 subunits and suppressed the observed wild-type aKv1/FL current by about 70% (Fig 5a & 5b). Similar results were also noted when the experiment was conducted in *Xenopus* oocytes following overnight light stimulation (S1 Fig), suggesting that such conditional mutant channels can be used in a variety of systems to down modulate Kv channels with light.

**Fig 6:**
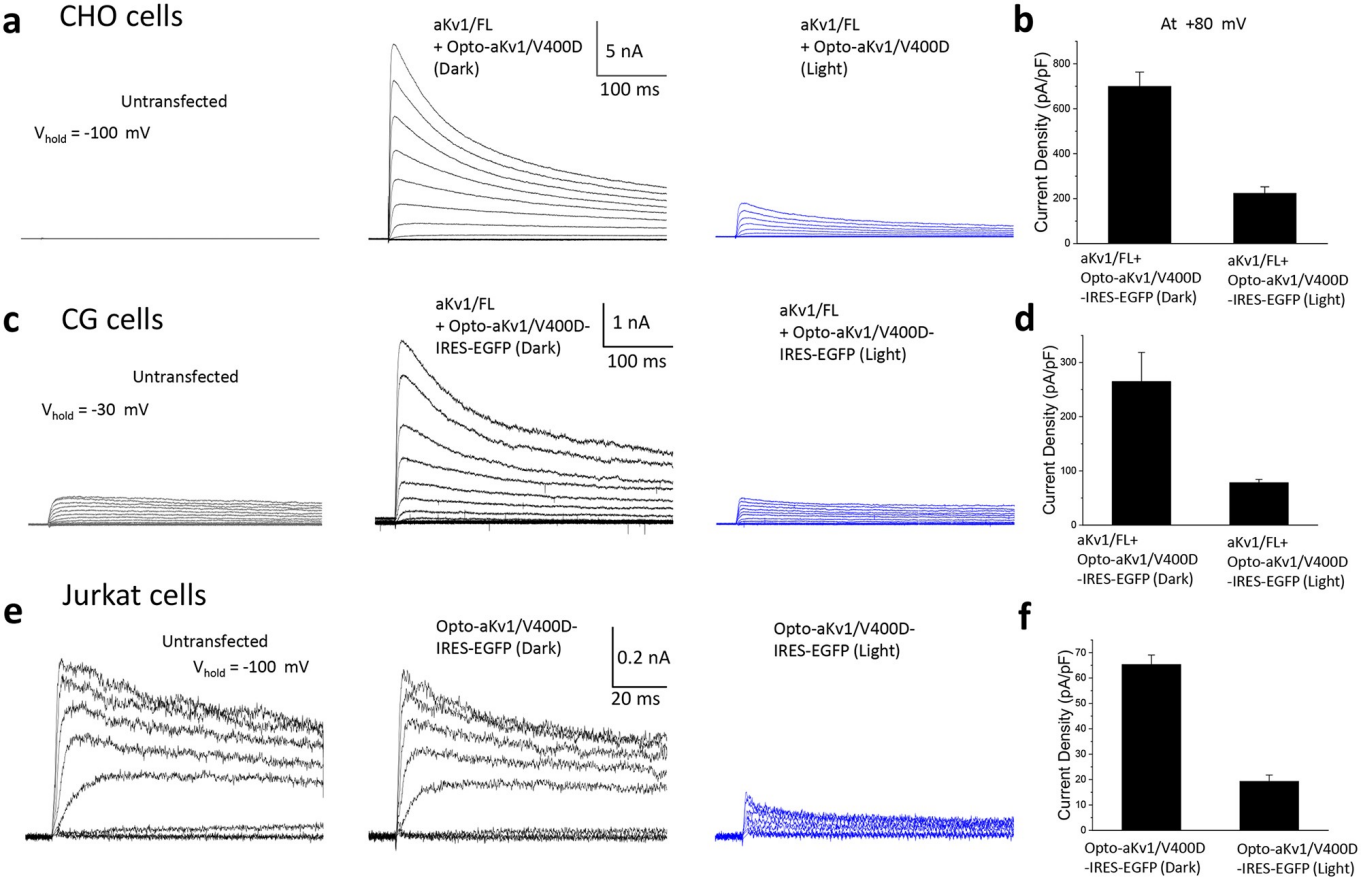
Photoactivation of dominant negative Opto-aKv1/V400D subunits in mammalian cells, cerebellar granule cells, and Jurkat cells. **a**) Photoactivation of Opto-aKv1/V400D markedly suppresses wild-type aKv1/FL current in CHO-K1 cells. CHO-K1 cells were co-transfected with wild-type aKv1/FL and Opto-aKv1/V400D, allowed to express overnight, and were either left in the dark or exposed to blue light for 1.5 hours before recordings. Whole-cell currents were elicited by step depolarization from -90 to +80 mV from a holding potential of -80 mV. **b**) Current density at +80 mV under dark and blue light conditions. Data is shown as mean ± SEM. Number of experiments: n = 12 for dark and n = 7 for blue light. **c**) Photoactivation of Opto-aKv1/V400D heterologously expressed in cerebellar granule (CG) cells also suppresses wild-type aKv1/FL current. To isolate the aKv1/FL current, the prominent endogenous Kv4 subthreshold A-current (I_SA_) was inactivated by a 900 ms-long prepulse to -30 mV before the test pulse to various potentials between +80 mV and -90 mV in 10 mV increments. Light treatment was the same as in A. **d**) Current density at +80 mV under dark (n = 8) and blue light (n = 4). **e)** Suppression of endogenous Kv1.3 in Jurkat cells by photoactivation of heterologously expressed Opto-aKv1/V400D channels. **f)** Current density of Kv1.3 current in Jurkat cells expressing Opto-aKv1/V400D in the dark (n = 4) and blue light (n = 5) (p < 0.01). Data is shown as mean ± SEM.

To determine if similar conditional dominant negative effects can be reproduced in neurons, we next electroporated wild-type aKv1/FL and Opto-aKv1/SL/V400D into dissociated cerebellar granule (CG) neurons and examined the currents with and without light exposure. Cerebellar granule neurons are small interneurons that express large subthreshold Kv4 based A-type currents (I-SA), as well as smaller non-inactivating K+ currents [36]. Because aKv1/FL functional properties are distinctive from any native CG currents, we could precisely measure the effectiveness of the light-regulated dominant native construct. For our measurements, the I-SA was easily separated from wild-type aKv1 by holding at -30 mV, a potential where aKv1 is not inactivated but Kv4 channels are, allowing us to focus on aKv1 currents. Recordings from CG cells showed similar responses as those observed in CHO cells (Fig 6c). When transfected CG cells were incubated in the dark, we detected large inactivating potassium currents, indicative of successful wild-type aKv1/FL expression. However, when exposed to blue light for 1.5 hours before recording, only small Kv currents were detected (Fig 6c). Photoactivation of Opto-aKv1/SL/V400D in CG cells resulted in an 80% reduction in current density, which was similar to the response magnitude observed in CHO cells (Fig 6d). However, the actual effect of current repression by Opto-aKv1/V400D is likely larger, since most of the remaining Kv current can be attribute to the smaller non-inactivating current (Fig 6c). Together, these data show that the dominant-negative Opto-Kv constructs are highly effective in knocking out compatible channels in neurons.

As members of the Kv1 subfamily, aKv1 subunits are expected to co-assemble with mammalian Kv1 subunits and form functional channels. To demonstrate that Opto-aKv1/V400D can produce dominant negative suppression of native Kv1 current upon blue light exposure, we heterologously expressed Opto-aKv1/V400D in Jurkat cells. Jurkat cells (Clone E6-1) are human T-lymphocytes that predominantly express Kv1.3 channels that plays a large role in controlling membrane potential. Whole-cell patch clamp recordings show that without expression of Opto-aKv1/V400D, Jurkat cells express endogenous outward potassium currents consistent with the reported Kv1.3 channels (Fig 6e). Expression of Opto-aKv1/V400D in the dark, verified by the expression of green fluorescent EGFP using an IRES construct, resulted in no significant change in the normal expression of Kv1.3 current (Fig 6e). However, after exposure to blue light for 1.5 hours, Jurkat cells expressing Opto-aKv1/V400D experienced a dramatic decrease in Kv1.3 current, showing dominant negative suppression of a native Kv1.3 current by photoactivated Opto-aKv1/V400D. Measurements of current density of Jurkat cells expressing Opto-aKv1/V400D confirms that the suppression is highly significant, and similar to what was observed in co-transfection studies (Fig 6f). Overall, our results demonstrate that the dominant negative construct Opto-aKv1/V400D can be used to suppressing native Kv1 channels upon photoactivation.

### Photoactivation of Kv1 dominant negative mutant in mitral cells increases sensitivity to novel odors

**Fig 7:**
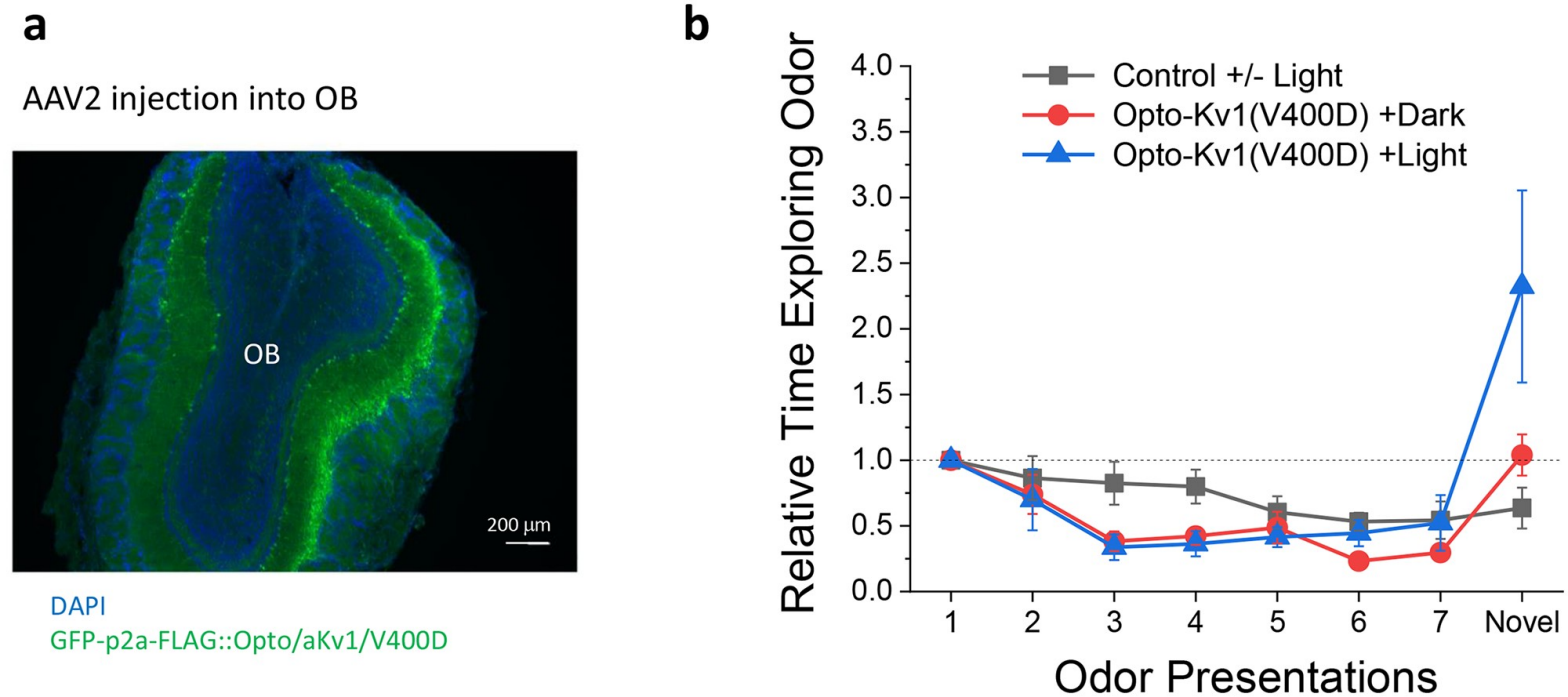
Photoactivation of Kv1 dominant negative mutant in mitral cells increases sensitivity to novel odor in mouse, similar to the supersmeller phenotype observed with Kv1.3 Knockout. **a**) Targeted expression of Opto-aKv1/V400D in olfactory lobe mitral cells via infections with the AAV-Ef1a-flex-EGFP-P2A-FLAG-OptoaKv1/V400D-WPRE virus. Green indicate the expression of the EGFP moiety that self-cleaves because of the P2A sequence. **b**) Photoactivation significantly increases the exploration time when a new odor is presented in the odor habituation test. The relative exploration time is plotted against the odor trial number, as the mice under different conditions were repeated exposed to the first odor at 1 min intervals than exposed to a second novel odor (See Materials & methods). On the eighth trial, the novel odor is presented to study discrimination against the first odor. (Data is shown as mean ± SEM. (Light/Control p<0.01; Dark/Control, N.S.).

To investigate the effectiveness of Opto-Kv channels to modify behavior following nervous system expression *in vivo*, we sought to alter the excitability of olfactory bulb mitral/tufted cells that play a key role in the encoding of odorant information [37]. Previous studies have found that mitral/tufted cells express Kv1.3 and that knockout of Kv1.3 produce changes in the neuronal excitability which alters behavior when tested in odor threshold and discrimination tasks [38]. To determine if we could induce a similar effect with light activation of Opto- aKv1/SL/V400D, we engineered adeno-associated viruses (AAVs) to conditionally express EGFP alone (AAV-Ef1a-flex-EGFP-P2A-FLAG-WPRE) or EGFP plus Opto-aKv1/V400D through a p2a linker (AAV-Ef1a-flex-EGFP-P2A-FLAG-OptoaKv1/V400D-WPRE) under the control of Cre recombinase. Transgene expression was selectively targeted to olfactory mitral/tufted cells by Flex switch activation in the transgenic mouse line Pcdh21-Cre, which selectively expresses Cre recombinase in olfactory bulb in mitral/tufted cells (Fig 7a) [39].

To activate Opto-aKv1/SL/V400D, experimental mice were stimulated immediately before habituation tests with blue light for 2–3 hours (per mammalian cell and neuron stimulation protocol) at 20 Hz, with 10 msec light pulses applied for 5 secs, followed by 25 secs with light off. This pattern was chosen to optimize LOV domain activation, while minimizing neuronal blue light exposure. The results showed that for control EGFP animals, repeated presentation of the first odor caused a gradual decrease in exploration time as mice became habituated to the odor, while the presentation of the novel odorant failed to elicit an enhanced response in the light or dark (Fig 7b). For mice expressing Opto-aKv1/V400D in the dark, they habituated normally, and showed a slight but not significant increase in exploratory time in response to the novel second odor (Fig 7b). Mice that were light stimulated had a robust 2.5 fold increase in exploratory time over the initial odor response (Fig 7b). These data indicate that selective photoactivation of Opto-aKv1/V400D in olfactory bulb mitral cells significantly increased the sensitivity of mouse to novel odors, similar to the behavioral phenotypes observed with Kv1.3 knockout [38].

## Discussion

**Fig 8:**
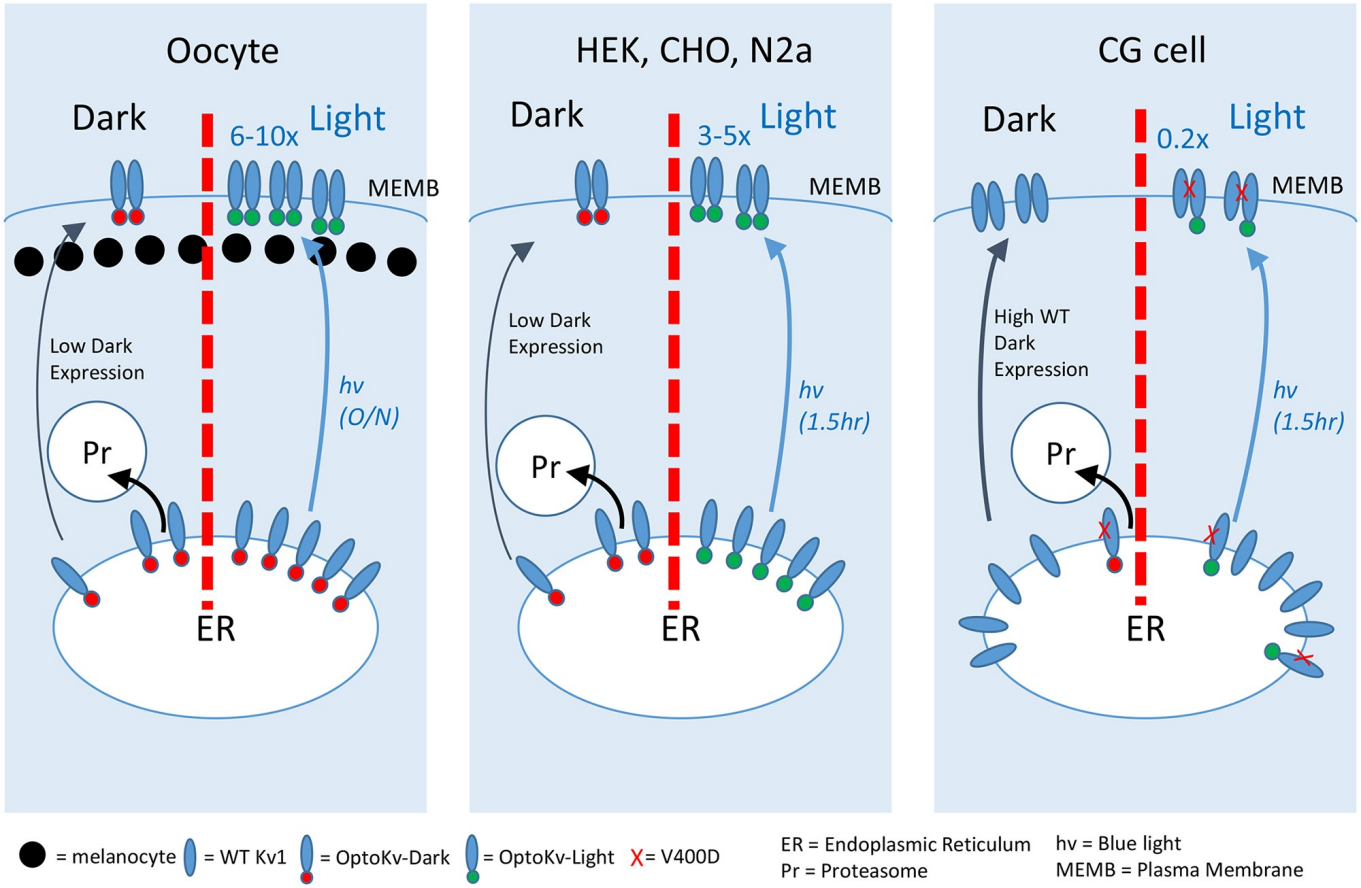
Expression of Opto-Kv channels in different expression systems. In the dark, Opto-Kv channel monomeric subunits are retained by the endoplasmic reticulum (ER) by trafficking machinery and shuttled for degradation because the inactive LOV domain (red) disrupts T1-dependent tetramerization and channel assembly. Upon blue light (hv) exposure, activated LOV do-main (green) frees the T1 domain to tetramerize, allowing fully assembled channels to easily traffic to the surface. **(left panel)** In Xenopus oocytes, blue light increases Opto-Kv current approximately 10-fold after overnight exposure at 18°C. **(center panel)** Blue light exposure for 1.5 hours is sufficient to rapidly increase surface expression about 3-5-fold by greatly increasing the fraction of tetrameric channels. This increase plateaus possibly due to limited subunit availability due to degradation of unassembled subunits prior to light exposure. **(right panel)** Neurons are making wild type channels but unassembled Opto-Kv-DN channels are shunted to degrade. Blue light treatment for 1.5 hours allows Opto-Kv-DN subunits to co-assemble with wild type dropping currents by 80%. Large effect is due to ability of one Opto-Kv-DN subunit to disrupt three wild type subunits.

In this paper, we have described the development and testing of a new optogenetic technique for regulating Kv channel expression by light. By fusing the light-sensitive LOV domain to the T1 tetramerization domain, we generated Opto-Kv channels that exhibit poor expression in the dark, but normal expression under blue light. The experimental results are consistent with a working model where the LOV domain in the dark state interferes with T1 domain tetramerization and promotes intracellular retention of the monomeric subunits. Photoactivation of the LOV domain frees the T1 domain to drive subfamily-specific assembly and produce channels that reach the cell surface (Fig 8). This on- and off-switch mechanism suggests that the observed time course is mainly a function of protein trafficking in agreement with the different kinetics for light-dependence in Xenopus oocytes and mammalian cells. As we have shown, this approach is not sensitive to the sequence placed N-terminal to the LOV domain, and indeed with aKv1 we find that there is normal N-type inactivation even with the LOV domain present. Moreover, we find that Opto-Kv channels with designer properties (i.e. altered inactivation, or dominant-negative suppression) can be used to decrease currents with light or test the importance of specific functional properties in a photoactivatable manner. This novel optogenetic tool will permit acute, light-dependent modulation of neuronal activity, and the evaluation of associated behaviors through genetically targeted regulation of Kv channels.

### LOV-T1 fusion and reduced expression

The fusion of a LOV domain to the N-terminus of T1 domain introduces a photoswitch that restricts channel surface expression in the dark and restores normal expression upon exposure to blue light. This was confirmed via electrophysiological recordings using multiple expression systems, immunocytochemistry, and biochemical-based biotinylation measurements. These new tools lend further support to the general design strategy of using LOV domains for affecting downstream elements. In our case, the downstream element is the T1 domain, a critical contributor to the assembly of Kv channels [6,10]. Although initially Aureochrome LOV domains were thought to specifically regulate N-terminal effectors, our work agrees with other studies that show that light-induced conformational changes in Aureochrome1 LOV domains occurs at both termini [27,40], and that N-terminal fusion of VfAU1/LOV to casp9 actually produced a larger effect than C-terminal fusion [28]. Interestingly, although the VfAU1/LOV domain was inserted downstream of the N-terminal inactivation domain in Opto-aKv1 channels, blue light stimulation did not produce a notable effect on inactivation kinetics for any of the constructs tested. This result agrees with earlier studies on aKv1 showing that charges on the N-terminus are more important for controlling N-type inactivation kinetics than the length of the “chain” [41,42].

Understanding the precise molecular mechanism by which the LOV domain negatively affects T1 tetramerization will require further structural studies. Previous studies on LOV domains have proposed various mechanisms of action, with the one thing in common being rearrangement in LOV secondary structure leading to allosteric effects on the effector domain [43]. With the LOV-Rac1 fusion construct, the LOV domain forms an extensive interface with Rac1 and prevents it from becoming active by an occlusion of the effector binding site of Rac1 [30]. Other popular modes of action propose photoactivation results in homo- or heterodimerization that eventually result in protein-protein interactions.

Importantly, in this study we also found that when the LOV domain is photoactivated, the Opto-Kv T1 domain is able to interact with wild-type Kv1 type T1 domain to form heterotetrameric channels. This is critically important since it is desirable for the Opto-aKv1/V400D subunits to tetramerize with wild-type Kv1 subunits to suppress Kv1 currents when the LOV domain is photoactivated. The robust current suppression of wild-type Kv1 channels by Opto-aKv1/V400D suggests that the T1 domain structure is mostly intact, and a simple light-dependent conformational change in the LOV domain can restore the Kv1 assembly identity.

### Cellular mechanism underlying the light-dependent activation and dark-dependent deactivation

We have also explored the cellular basis for the reduction in surface expression in the dark and the restoration upon exposure to blue light. In the dark, the LOV domain disrupts T1-mediated channel assembly and, as immunocytochemistry and surface biotinylation data show, traps the unassembled subunits inside the cell (Fig 5). It is likely that the unassembled subunits are retained in the endoplasmic reticulum, which acts as a quality control checkpoint for proteins heading for the surface. Proteins retained in the ER are often subject to degradation by processes such as proteasome-mediated protein degradation, which may be occurring at varying degrees. An unanswered question is whether the pool of retained, monomeric Opto-Kv1 subunits are the primary source of the newly formed surface channels upon photoactivation, or whether the surface channels primarily come from newly synthesized subunit proteins. In cultured neurons we found enhanced effects by including lactacystin in the culture media, which may act by both slowing the breakdown of unassembled Opto-Kv1 channels in addition to up-regulating antioxidant proteins as a defense against light generated reactive oxygen species [44]. We are testing additional ways to manipulate the breakdown of unassembled Opto-Kv1 subunits as well as blocking new protein synthesis to address this question.

### Use of Opto-Kv channels in neurons and *in vivo*

The large number of different potassium channel variants expressed in the central nervous and the strong homeostatic regulation of neuronal excitability creates tremendous difficulties in probing the function of different classes of ion channels in different cell types. Typically these studies either involve pharmacological agents, which is poorly localized, or they require a considerable time between the genetic manipulation and the functional/behavioral assay, which means that the homeostatic regulatory systems have likely produced significant changes in function during the lag [45,46]. Despite this, many intriguing roles for potassium channels and their interactions with other regulatory proteins have been identified that are likely critical for normal neuronal function and may underlie neurological and psychiatric disorders [47–52]. Our results show that Opto-Kv channels are an important new tool in probing the behavioral roles of ion channels in specific neuronal populations. Light-induced expression of dominant-negative Opto-Kv1/V400D channels in mitral cells of the olfactory bulb results in behavioral changes consistent with Kv1.3 knockout. Odor habituation experiments tested discrimination between 1-heptanol and 1-octanol, two molecules with only a single carbon chain length difference, and photoactivation of Opto-aKv1/V400D dominant negative subunits increased odor exploration time by 2.5-fold. Our results compare favorably with the previous finding on alcohol discrimination by Kv1.3 knockout mice, where exploration time ratio increased less than 1-fold between ethyl butyrate, and approximately 2-fold between ethyl heptanoate and ethyl caprylate [38]. Some reports have shown a great variability in the turnover of neuronal proteins depending on the protein identity, cell type, tissue of origin, age of animal, and whether the neurons are mixed with glia cells [53,54]. Therefore, it is possible that different optimization parameters will be needed to use these constructs in other neuron types, or expression systems, but thus far conditions optimized in CHO cells have translated well to neurons.

### Final remarks

This work presents a novel optogenetic tool available to change the functional levels of Kv channels in cells by light. Traditional methodologies used to alter Kv channel functional levels consists of selective pharmacological blockers, transcriptional regulators (i.e. gene knockout and RNA interference), and heterologous expression using constitutive or inducible promoters. Each method has its distinct advantages and disadvantages. A more recent approach using cysteine-modified Kv channels and photoactivatable quaternary ammonium blockers provides fast and reversible light-induced regulation of Kv currents, but the approach presents many technical challenges for in vivo experiments because of background native channels [5,55]. While all these approaches have their utility, the Opto-Kv technology offers a combination of relatively easy implementation, channel specificity, cell-specific targeting, and reversibility.

The advantages of channel specificity and cell-specific targeting comes with certain disadvantages. Our Opto-Kv dominant negative approach depends on the Kv T1 domain for both specificity and control of assembly and surface expression; therefore, the approach is limited to Kv channels and differential effects are likely to occur depending on heteromultimerization with different subfamily isoforms expressed in the examined experimental system. Furthermore, an important consideration in implementing studies using Opto-Kv channels should be an evaluation of the rates of forward protein trafficking and protein recycling, since they may vary between cell types. Finally, additional optimization of the photocycle may be possible through mutational analyses that alters the LOV domain to favor the cysteinyl-flavin adduct (LOV390) state or the relaxed dark state (LOV447) [56].

In summary, we have developed a novel set of Opto-Kv channels using a strategy that works on Kv channel assembly via the T1 domain. Further research may allow this LOV-based strategy to be applied to other channel assembly domains, opening up the possibility of optogenetic regulation of additional native channels. Our results show that this strategy can effectively create light-sensitive channels with native or altered properties, and the implementation of dominant-negative Opto-Kv constructs was shown to produce light-induced transient knockout of native Kv channels. Using a similar approach, Opto-Kv channels may be useful to study disease-causing mutations in Kv channels in a reversible way, by up and down regulating the mutant subunit in the same animal using light.

## Materials and methods

### Ethics statement

The animal procedures conducted in this work were performed in strict accordance with the Animal Welfare Act, the Public Health Services Animal Welfare Policy, and The National Institute of Health Guide for Care and Use of Laboratory Animals. The experimental protocol was approved by the Institutional Animal Care and Use Committees (IACUC) of Baylor College of Medicine (Protocol Numbers: AN-752, AN-4632). Following the approved protocol, every effort was made to minimize suffering.

### Molecular biology

To create Opto-AKv channels, synthetic gene synthesis (CelTek Genes Service) was used to construct a VfAu1 LOV domain sequence codon optimized for human (Fig 1b) with a small fragment of the aKv1 channel sequence included at the C-terminus to allow for in-frame fusion with aKv1 at a unique Bcl1 site. Functional variants of the Opto-aKv1 construct with shortened N-terminal chain sequences (Medium: MEVAMAGIEGNGGPAGYRDSYHSSQRPLLRSSNLPNSRSFP; Short: MEVAMAGIEGNGGPAGYRDSYHSSQRP) were also made using synthetic gene synthesis. Opto-rKv1.1, Opto-rKv2.1, and Opto-rKv3.1 were designed homologously to Opto-aKv1 and made by first using overlap extension PCR to create respective VfAu1/LOV-T1 domain fragments with the necessary flanking sequences and then subcloning them back into the respective channel constructs. A dominant negative Opto-aKv1 construct containing the pore mutation V400D (aKv1 version of Drosophila Shaker mutant Sh^KS133^) was made by subcloning relevant portions of aKv1(V400D) (previously made using QuickChange mutagenesis (Agilent)) into Opto-aKv1. All constructs were fully sequenced over the open reading frame for verification, and cRNAs for oocyte injection were prepared by linearization of constructs followed by transcript synthesis in vitro using T7 or T3 MessageMachine kits (Ambion).

The following constructs were generated for expression in mammalian cells and neurons. pCMV/aKv1.1/FL, pCMV/OptoaKv1/ML, pCMV/OptoaKv1/SL, pCMV/Opto-aKv1/V400D constructs were generated by inserting the relevant ORF from the oocyte constructs into a pCMV vector via subcloning. To increase the likelihood that EGFP-expressing neurons would also express dominant negative constructs, a multicistronic construct was generated (pCMV/Opto-aKv1/V400D/IRES/EGFP) where Opto-aKv1/V400D and EGFP would be translated off of a single mRNA transcript. For gene delivery and expression in olfactory bulb, adeno-associated viruses (AAV-Ef1a-Flex-EGFP-P2A-FLAG-WPRE and AAV-Ef1a-Flex-EGFP-P2A-FLAG-OptoaKv1/V400D-WPRE) were generated by inserting Opto-aKv1/V400D into the appropriate viral vector construct.

### Light induction

We performed light stimulation using a calibrated array of 10 royal blue LEDs (465–470 nm) running for variable lengths of time depending on the experiment. Super Bright Blue 5mm LEDs (Adafruit) were purchased from a local computer and electronics retailer (Micro Center), and each LED produces a brightness of 6000 millicandelas with 20 degrees spread at a suggested current of 30 mA. Powered by a consumer-grade 9-volt battery, our array of 10 LEDs carried a current of 5 mA, resulting in each LED producing 0.095 lumens per 1.13 cm^2^, or a calculated 14.2 μW/mm^2^. For some experiments, the current was increased to 10 mA to raise the brightness to 28.4 μW/mm^2^. In addition, a royal blue LED coupled to a light guide (BioLED, Mightex) was added to our oocyte physiology rig to test for acute functional regulation of channels by light. At 100%, the blue LED used for acute exposure produced a brightness of 12.2 μW/mm^2^, which is comparable to the brightness used for long-term exposures.

### Oocyte harvest and heterologous expression in *Xenopus* oocytes

*Xenopus laevis* frogs (Nasco) were anesthetized with 0.1% Tricane solution (Sigma) absorbed through the skin, and stage V–VI oocytes were surgically harvested and defolliculated by collagenase I treatment. Oocytes were injected with cRNAs using a Nanoinjector (Drummond Scientific Company). Injected oocytes were incubated at 18°C for 1–2 days in standard ND96 solution (in mM: 96 NaCl, 2 KCl, 1.8 CaCl_2_, 1 MgCl_2_, and 5 HEPES, pH 7.4 adjusted with NaOH) supplemented with 5 mM Na-pyruvate and 5 mg/ml gentamycin.

### Heterologous expression in cultured mammalian cells

Human embryonic kidney (HEK293T) cells were cultured in DMEM+GlutaMAX^™^-1 (Dulbecco’s modified Eagle’s medium plus 4.5 g/L D-glucose and 110 mg/L sodium pyruvate; Invitrogen) supplemented with 10% fetal bovine serum (FBS; Invitrogen), 100 units/ml penicillin, and 100 units/ml streptomycin at 37 °C in humidified 5% CO_2_ atmosphere. Chinese Hamster Ovary (CHO-K1) cells were cultured in F12 media with 10% FBS, 1x GlutaMax, and 1x Pen/Strep. Jurkat (E6.1) cells were cultured in ATCC-formulated RPMI-1640 medium supplemented with 10% FBS and 1x Pen/Strep (Invitrogen). As suspension cells, Jurkat cells were split every 2–3 days and kept at between 1 x 10^5 to 1 x 10^6 cells/ml. For patch clamp recordings, cells were plated on 12-well clusters at 45% seeding density and transfected the following day using Lipofectamine 2000 following manufacturer’s instructions. After successful transfections were verified, the cells were re-plated on glass coverslips previously coated with poly-D-lysine (PDL; Sigma) at 25% density to give 50% density on the day of the recording. Jurkat cells were electroporated with DNA using NEON transfection system (Invitrogen) as according to company protocol, and on the next day the cells were plated on PDL-precoated coverslips (Neuvitro Corporation) and allowed to attach for 30–60 min before patch recording.

### Heterologous expression in cerebellar granule cells

Preparation of dissociated CG cells has been previously described. Briefly, glass coverslips were first coated with PDL (0.1 mg/ml in H2O), washed 3x with water, and then coated with laminin in HBSS without Mg or Ca (100 ul in 10 ml). Coverslips were incubated overnight in 37 °C incubator then rinsed 3x with DPBS for 10 min each time. P2-P4 mouse pups were decapitated following isofluorane anesthesia, and their brains were removed. The cerebella were dissected and minced using a sharp blade on Whatman filter wet with HBSS without Mg and Ca, and the brain material was placed in 1.5 ml microcentrifuge tubes and spun down at 200 x g on tabletop centrifuge for 5 min. After removing the supernatant, 300 ul of trypsin-EDTA was added per tube, and the tubes were incubated in a 30°C waterbath for 15 min with mixing every 5 min. After a quick spin, the trypsin was removed and replaced with DNase I (125 ug/ml) in HBSS with Ca and Mg (HBSS/CM). The cerebellar material was triturated 3x using 1 ml pipette tips. After the material was allowed to settle, the supernatant with dissociated cells in suspension was pipetted into a new tube and then spun down again. The cells were then resuspended in 1 ml of plating medium without antibiotics (in MEM: 10% FBS. 1x B-27 supplement, 1x Pen/Strep, 1x GlutaMax, 20 mM KCl). The CG cells were counted and diluted to appropriate concentration for electroporation using NEON.

DNA constructs were electroporated into CG cells following NEON transfection system protocol (Invitrogen). Prepared CG cells were washed with PBS/CM, spun down, and resuspended in Resuspension Buffer R at 1x10^7 cells/ml. The cells were pipetted up to a NEON tip of appropriate size and electroporated at the following settings: pulse voltage = 1500 V, pulse width = 10 ms, and pulse number = 1. Cells were then plated out on coverslips placed in 24-well cluster with plating media, and on the following day, the plating media was replaced with Neurobasal growth media supplemented with 1x B-27, 1x pen/strep, 1x GlutaMax, and 20 mM KCl. Patch recordings were conducted on CG cells within about 3–7 days after plating. We found that CG neurons in culture were sensitive to sustained blue light exposure, therefore, to enhance cultured neuronal health, we switched to LiveLight NEUMO photostable medium (Cell Guidance Systems) supplemented with proteasome inhibitor lactacystin (10 uM; Sigma) overnight prior to blue light treatment. Neuronal health and transfection efficiency were monitored by co-expression of EGFP, as well as verifying normal electrophysiological properties.

### Viral Injection for expression in olfactory bulb

AAV-Ef1a-Flex-EGFP-P2A-FLAG-WPRE and AAV-Ef1a-Flex-EGFP-P2A-FLAG-OptoaKv1/V400D-WPR (Serotype DJ/8) were packaged and titered by the Optogenetics and Viral Vectors Core at the Jan and Dan Duncan Neurological Research Institute. Viral particles were injected via stereotaxic injection into the main olfactory bulb (from bregma: ML, +0.9 mm; AP, 3.82 mm, and 0.9 mm from bulb surface) of Pcdh21-Cre(+/-) mice using glass injection needles and a Nanoject II (Drummond Scientific Company, Broomall, PA). The injection was conducted at a rate of 63 nl/sec at 20 second intervals to obtain uniform labeling.

### Two-electrode voltage clamp (TEVC) and whole-cell patch clamp electrophysiology

TEVC recordings were performed in *Xenopus* oocytes using two microelectrode voltage clamp as described in our published protocols [57]. The microelectrodes had <1 MOhm tip resistances and were filled with 3 M KCl solutions. The voltage-clamp amplifier (Oocyte Clamp OC-725C, Warner Instruments) was under the control of WinWCP software (John Dempster, University of Strathclyde, Glasgow, UK). The data were digitized and low-pass filtered (model 902, Frequency Devices) at various frequencies depending on the sampling rate. The capacitive transient and linear leak subtraction were performed off-line. Recordings with offsets >3 mV were removed from data analysis, and the average leak current was <0.2 mA.

Mammalian cells and CG neurons were visualized using a fixed-state upright microscope (BX51WI, Olympus) equipped with 5x and 40x UMPlanFI objectives. Patch pipettes were fabricated by pulling standard borosilicate glass capillaries (1B150-4, World Precision Instruments) on a Flaming/Brown micropipette puller (model P-97, Sutter Instrument Co.). Pipette tip resistance was typically between 2–5 megaOhm. A MultiClamp 700B (Axon Instruments) was used to deliver voltage pulses under the control of MultiClamp Commander and pClamp10 (Axon Instruments) with the assistance of Digidata 1550 low-noise data acquisition system (Axon Instruments). The data were acquired on a desktop personal computer.

Tight-seal whole-cell recordings were obtained using standard patch clamp techniques. For cultured cells recordings, the patch electrodes were backfilled with a solution containing (in mM): 140 KCl, 1 CaCl_2_, 1 MgCl_2_, 10 EGTA, 10 HEPES (pH 7.4), and 5 Glucose; the bath solution contained (in mM): 140 NaCl, 5 KCl, 2 CaCl_2_, 2 MgCl_2_, 10 HEPES (pH 7.4), and 10 Glucose. For CG cell recordings, the patch electrodes were backfilled with a solution containing (in mM): 120 K-gluconate, 20 KCl, 4 NaCl, 10 HEPES (pH 7.25), 4 Mg-ATP, 0.3 Na-GTP, 7 Phosphocreatine, and the bath solution contained (in mM): 124 NaCl, 4.4 KCl, 0.1 CaCl_2_, 3.1 MgSO4, 1 NaH2PO4, 0.1 4-AP, 0.0005 TTX, 26 NaHCO3, and 10 Glucose. Series resistance and whole-cell capacitance were determined by optimal cancellation of the capacitive transient. The series resistance was typically 6–10 megaOhm, about 2x the tip resistance. Series resistance compensation was typically around 75–80%. The data has not been corrected for liquid junction potential between the electrode and bath solution (about -14.2 mV calculated). All experiments were conducted at room temperature (22–23°C). Ohmic leak were subtracted off-line.

### Protein biotinylation

CHO-K1 cells were plated on 12-well clusters and transfected precisely following Lipofectamine 2000 protocol (Invitrogen). On the day following transfection, Sulfo-NHS-SS-Biotin (Sigma) was dissolved in DPBS/CM (138 mM NaCl, 2.7 mM KCl, 8 mM Na2HPO4-7H2O, 1.5 mM KH2PO4, 1 mM CaCl_2_, 0.5 mM MgCl_2_-6H2O, pH 7.4; Thermo Fisher Scientific, #14040) at 0.5 mg/ml and stored on ice. The cells were washed with 1 ml of ice-cold DPBS/CM twice. Afterwards, 0.3 ml of the Sulfo-NHS-SS-biotin solution was added, followed by incubation on ice for 30 min with occasional shaking. The cells were then washed three times with ice-cold quenching solution (50 mM glycine in PBS/CaCl_2_/MgCl_2_, pH 7.4), and then they were lysed with 120 ul of immunoprecipitation (IP) buffer (150 mM NaCl, 50 mM Tris-HCl, 1% Triton X-100, 0.1% SDS, and protease inhibitor) and incubated 10 min on ice. The lysates were transferred to 1.5-ml tubes and spun at Vmax (about 14,000 rpm) on a 4°C desktop centrifuge for 10 min to remove cellular debris. A small aliquot (10 ul) of the supernatant was saved for later analysis, and the rest was mixed with 50 ul of 50% slurry of equilibrated streptavidin-agarose beads (Thermo Fisher Scientific) and incubated overnight at 4°C with constant mixing. Next day, the beads were extensively washed 3x with 1 ml of lysis buffer by centrifugation at 500 x g for 1 min. After the final wash, the supernatant was removed, and 50 ul of 2x Laemmli sample buffer was added (2x sample buffer: 100 mM Tris-HCl, 200 mM dithiothreitol, 4% SDS, 0.2% bromophenol blue, 20% glycerol, pH 6.8). The beads were then boiled at 100°C for 5 min to elute the bound proteins, which were loaded on SDS-PAGE for Western analysis.

### Immunocytochemistry

Cells transfected with HA-OptoaKv1 or HA-WT were exposed to blue light for 1.5hrs at 37 °C, then washed 1x with PBS, fixed with 4% paraformaldehyde in PBS for 10min at room temperature (RT), then washed 3x with PBS for 5min each. Cells were blocked with 10% goat serum for 1 hr at RT, with 0.2% tritonx-100 if permeabilized. Rat anti-HA primary antibody at 1:500 (Roche) was incubated overnight in blocking solution at 4C. Following 3x 5min washes in blocking solution at RT, coverslips were fluorescently labeled with Alexa-Fluor conjugated goat anti-rat at 1:1000 (Invitrogen) for 1hr in blocking solution, then washed 3x for 10min with PBS and mounted on microscope slides using DAPI Fluoromount-G mounting media (SouthernBiotech). Images were acquired with AxioImager Z1 microscope (Zeiss) using appropriate filters and analyzed using ImageJ.

### Behavioral study in mouse

Transgenic mice (Pcdh21-Cre), in which protocadherin 21 promoter drives the expression of Cre recombinase, were injected at the 12 weeks of age with AAV-Flex virus expressing either EGFP alone (AAV-Ef1a-Flex-EGFP-P2A-FLAG-WPRE) or with Opto-aKv1/V400D (AAV-Ef1a-Flex-EGFP-P2A-FLAG-OptoaKv1/V400D-WPRE). About 690 nl of virus (2–4 x 10^11 MOI) was injected into each olfactory bulb, and behavioral tests were performed 4 weeks after injections. For experiment in freely moving mice, a cannula guide is pre-fixed to the skull by dental cement over the location of the injections to allow attachment of the optic fiber. Two days prior to the tests, the animals were individually habituated to a new cage for 10 minutes daily for 2 days. On the day of the experiment animals were tethered to a fiber optic cable. During the experiment the investigator was blinded to the genotype of the animal. Mice were stimulated by blue light for 90 minutes using 10 msec light pulses running at 20 hz, with 5 secs on and 25 secs off. A DoricLens blue laser (465 nm wavelength, ~4mW) was used. The odor habituation test was based on the approach of Fadool et al, 2004 and consisted of each mouse performing 8 1-minute odor exposure trials with 1 minute of rest between each trial [38]. For trials 1 through 7, 1-heptanol (diluted 1:100) was placed on a Q-tip, and the mouse investigation time was recorded. For trial 8, the odor was switched to 1-Octanol (diluted 1:100), and the mouse investigation time was once again recorded. Once all the measurements were completed, the investigator was unblinded and exploration time was normalized to trial 1 for each animal. The average normalized time for each group is plotted with error bars denoting SEM.

### Data analysis

Data were analyzed with WinWCP (John Dempster, University of Strathclyde, Glasgow, UK) and Origin software (OriginLab Corp.). Significance testing was performed using an independent t-test with significance levels set at 0.05 after verification that the measurements show Gaussian scatter [58].

## Supporting information

S1 Fig Light-activated suppression of aKv1/FL current by dominant negative Opto-aKv1/V400D construct in oocytes. **a)** Currents by oocytes expressing aKv1/FL and Opto-aKv1/V400D incubated overnight in the dark or under blue light. Currents were elicited by 500 ms step depolarizations from -100 mV holding potential in 10 mV increments under elevated external K+ condition. **b**) Measurements of peak current at +50 mV under dark (n = 3) and light conditions (n = 4) (p < 0.01). Mean ± SEM.
(TIF)
