# Supplementary figures and images for: Light-regulated voltage-gated potassium channels for acute interrogation of channel function in neurons and behavior
Source: PLoS One. 2021 Mar 23;16(3):e0248688. doi: 10.1371/journal.pone.0248688 (PMC7987177; doi:10.1371/journal.pone.0248688)

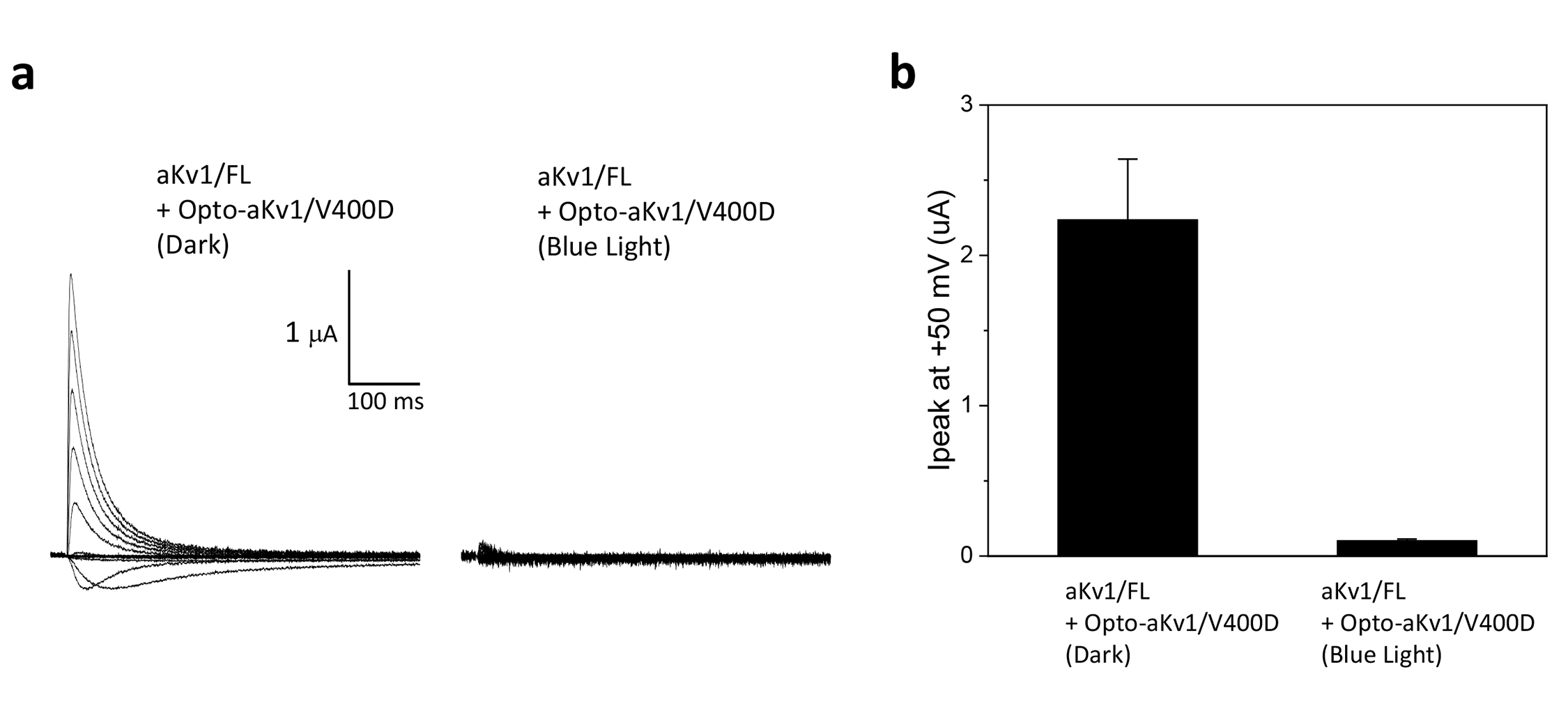

Supplement: S1 Fig — a) Currents by oocytes expressing aKv1/FL and Opto-aKv1/V400D incubated overnight in the dark or under blue light. Currents were elicited by 500 ms step depolarizations from -100 mV holding potential in 10 mV increments under elevated external K+ condition. b) Measurements of peak current at +50 mV under dark (n = 3) and light conditions (n = 4) (p < 0.01). Mean ± SEM. (TIF) [file pone.0248688.s001.tif]
